# Clinical Management of Patellar Tendinopathy in Athletes: A Narrative Review of Diagnosis, Treatment, and Return to Sport

**DOI:** 10.3390/healthcare14193248

**Published:** 2026-10-01

**Authors:** Yuhao Chen, Xin Kuang, Xinyan Zheng

**Affiliations:** 1 School of Exercise and Health, Shanghai University of Sport, Yangpu, Shanghai 200438, China; 23600504@sus.edu.cn (Y.C.); 2421518038@sus.edu.cn (X.K.); 2 Sports Medicine and Rehabilitation Center, Shanghai University of Sport, Shanghai 200438, China; 3 Shanghai Shangti Orthopaedic Hospital, Shanghai 200438, China

**Keywords:** patellar tendinopathy, diagnosis, exercise therapy, prognosis, clinical management

## Abstract

**Highlights:**

**What are the main findings?**

- Current evidence does not establish the superiority of any single exercise loading mode. Evidence for injection therapies varies across intervention types and is derived mainly from studies with short-term follow-up; whether any injection provides a sustained benefit beyond concurrent exercise remains uncertain, and current evidence does not support the use of extracorporeal shockwave therapy as a first-line or stand-alone intervention.
- Structural imaging findings may be associated with subsequent symptom development in selected populations, but their incremental value beyond clinical assessment remains uncertain. Imaging findings should therefore be interpreted alongside symptoms and function when assessing severity, treatment response, or return-to-sport readiness.

**What are the implications of the main findings?**

- Clinicians may consider loading-based rehabilitation as the principal conservative approach, while recognizing that the underlying evidence is of low to very low certainty and does not establish the superiority of any single loading mode.
- Load progression and return-to-sport decisions should be driven by functional outcomes (pain, function, and patient-reported recovery) rather than by imaging findings.

**Abstract:**

Patellar tendinopathy affects approximately 18% of athletes and up to 45% of volleyball players, representing a common cause of load-related anterior knee pain and functional limitation in jumping and landing sports. Clinical diagnosis relies primarily on history and physical examination. However, no consensus has yet been established on standardized diagnostic criteria, particularly for imaging-based assessment, and no definitive clinical practice guideline currently exists; imaging abnormalities frequently coexist with symptoms but are also common in asymptomatic athletes, and incidental findings may carry a risk of overdiagnosis and unnecessary imaging-guided management. This narrative review synthesizes the current evidence on the clinical management of patellar tendinopathy in athletes, spanning diagnosis, treatment, long-term prognosis, and return to sport; the literature was identified through structured searches of PubMed, Web of Science, and Google Scholar, with the final search conducted on 1 July 2026. Exercise therapy represents the most extensively studied conservative approach and may be considered a reasonable first-line option, although no single loading mode has demonstrated clear superiority and the underlying evidence is of low to very low certainty; injection strategies differ in mechanisms and evidence strength, but most data come from short-term studies. These studies commonly combine injections with exercise, making the injection’s isolated effect unclear. Additionally, shockwave therapy is not supported by current evidence as a first-line or stand-alone option. Because structural imaging changes and clinical symptoms frequently diverge, functional outcomes rather than imaging endpoints should guide load progression and return-to-sport decisions. This review provides clinicians with a practical synthesis of the currently available evidence for treatment selection and patient management, and identifies remaining uncertainties, particularly in adolescent populations and long-term outcome prediction.

## 1. Introduction

Patellar tendinopathy is among the most common and debilitating overuse injuries in jumping and landing sports, presenting with load-related anterior knee pain and functional limitation that may persist for months to years. The overall prevalence among athletes is approximately 18% [1], rising to 44.6% in elite volleyball players [2]. The long-term burden is considerable: a 15-year follow-up reported that over half of athletes with a history of jumper’s knee retired from sport due to knee problems [3]. The condition spans the full athletic spectrum, from elite performers to recreational participants, and its impact extends beyond pain to include recurrent disability, time loss from training and competition, and a substantial personal and economic cost.

Clinical decision-making in patellar tendinopathy is complicated by several unresolved clinical questions. Diagnosis rests primarily on history and physical examination; no universally accepted diagnostic gold standard exists, and structural imaging findings frequently diverge from symptoms. Exercise-based rehabilitation is generally considered a key component of management, yet trials report heterogeneous results across loading modes, and the role of adjunctive interventions such as injections and physical modalities remains debated. Long-term outcomes are likewise uncertain: recovery trajectories vary widely, objective criteria to guide return to sport (RTS), the process of resuming sport participation after injury, viewed here as a continuum of return to participation, sport, and performance rather than a single decision point [4], are lacking, and most prognostic data derive from discontinuous follow-up. These gaps translate directly into difficulty in treatment selection, load progression, and rehabilitation planning for the individual athlete.

Existing reviews have addressed prevalence, risk factors, diagnosis, and treatment largely as separate domains: recent evidence syntheses have focused on management [5], on sex-specific data gaps [6], and on outcome measurement [7,8], whereas the treatment reviews most often used as clinical guidance, such as those by Malliaras et al. [9,10], predate the landmark trials on progressive tendon loading and loading parameters of recent years [11,12,13,14] and do not reflect the updated treatment syntheses [15,16] or the recent prognostic evidence, including the first recovery prediction model for the condition [17]. Three areas remain only partially addressed in the existing literature. First, evidence on adolescent populations and long-term prognostic trajectories has not been integrated into a single management framework, even though these populations pose the most demanding clinical questions. Second, most prior reviews adopted a 2021 cutoff, and several relevant studies on prognostic factors and physical modalities have since been published. Third, although imaging-symptom discordance has been recognized, it is rarely examined as a cross-cutting consideration in management decisions rather than as an isolated diagnostic puzzle. The present review addresses these gaps by integrating evidence published since 2021 across diagnosis, treatment, prognosis, and RTS, and by examining imaging–symptom discordance as a decision-relevant dimension with practical clinical implications, including screening, incidental findings, and the risk of overdiagnosis.

## 2. Literature Search Strategy

This narrative review was informed by a structured search of PubMed and the Web of Science Core Collection, supplemented by Google Scholar and reference-list screening. Studies published from January 2010 to 1 July 2026 were considered. Earlier influential epidemiological studies, cohorts, clinical models, and trials were retained when identified from the reference lists of included studies and reviews. The search covered four predefined domains: epidemiology and risk factors, diagnosis and imaging, treatment and rehabilitation, and prognosis and return to sport.

The exact final search strings used in each database were as follows. In PubMed, quoted terms were searched in all fields: (“patellar tendinopathy” OR “patellar tendinitis” OR “patellar tendonitis” OR “jumper’s knee”) AND (“prevalence” OR “incidence” OR “epidemiology” OR “risk factor” OR “diagnosis” OR “ultrasound” OR “ultrasonography” OR “magnetic resonance imaging” OR “MRI” OR “structure” OR “treatment” OR “rehabilitation” OR “exercise” OR “eccentric” OR “isometric” OR “heavy slow resistance” OR “HSR” OR “loading” OR “shockwave” OR “extracorporeal shockwave therapy” OR “platelet-rich plasma” OR “PRP” OR “plasma rich in growth factors” OR “dry needling” OR “corticosteroid” OR “injection” OR “hyaluronic acid” OR “polidocanol” OR “surgery” OR “taping” OR “glyceryl trinitrate” OR “GTN” OR “blood flow restriction” OR “prognosis” OR “return to sport” OR “return to play” OR “follow-up” OR “long-term outcome”). In the Web of Science Core Collection, the same terms were applied to the topic field (title, abstract, and keywords), with the search restricted to publication years 2010–2026: (“patellar tendinopathy” OR “patellar tendinitis” OR “patellar tendonitis” OR “jumper’s knee”) AND (“prevalence” OR “incidence” OR “epidemiology” OR “risk factor” OR “diagnosis” OR “ultrasound” OR “ultrasonography” OR “magnetic resonance imaging” OR “MRI” OR “structure” OR “treatment” OR “rehabilitation” OR “exercise” OR “eccentric” OR “isometric” OR “heavy slow resistance” OR “HSR” OR “loading” OR “shockwave” OR “extracorporeal shockwave therapy” OR “platelet-rich plasma” OR “PRP” OR “plasma rich in growth factors” OR “dry needling” OR “corticosteroid” OR “injection” OR “hyaluronic acid” OR “polidocanol” OR “surgery” OR “taping” OR “glyceryl trinitrate” OR “GTN” OR “blood flow restriction” OR “prognosis” OR “return to sport” OR “return to play” OR “follow-up” OR “long-term outcome”).

The searches identified 754 records in PubMed and 915 in Web of Science. After removal of 555 duplicates, 1114 unique records underwent title and abstract screening. A separate grey literature search was not conducted.

Human cohort studies, clinical trials, cross-sectional studies, systematic reviews, and meta-analyses directly addressing the predefined domains were considered. Studies unrelated to patellar tendinopathy, lacking human data, or not relevant to the clinical questions were excluded. Athlete-specific evidence was prioritized, while relevant non-athlete evidence was retained when athlete-specific evidence was limited. Adolescent evidence was considered separately. Of the 1114 unique records screened at the title and abstract level, 156 records were assessed in full text. After full-text assessment, 86 records were excluded, mainly because they did not meet the predefined inclusion criteria. The remaining 70 publications were included. Google Scholar was used separately as a supplementary source to the database searches and the first 100 results ranked by relevance from each of four domain-specific searches were examined. In addition, the reference lists of the included studies and reviews were screened to identify influential foundational studies, and a further 12 publications were identified through these supplementary approaches. All 12 were assessed in full text against the same criteria and were included for citation. As the evidence base underlying the clinical recommendations includes studies and evidence syntheses that do not report athlete-specific subgroup results, the contribution of non-athlete evidence could not be reliably quantified. Owing to this objective limitation, recommendations informed by non-athlete or mixed-population evidence should be interpreted with caution. One reviewer performed the initial selection, a second reviewer checked the selected studies, and uncertainties were resolved through discussion and consensus.

Findings were synthesized qualitatively across the four domains. Evidence syntheses were prioritized, with individual primary studies used when they provided newer or additional information not covered by those syntheses. The review cited 82 publications, including four influential studies published before 2010. No formal risk-of-bias assessment was performed; therefore, incomplete retrieval and selection bias cannot be excluded, and the conclusions should be interpreted according to the design and consistency of the available evidence.

## 3. Epidemiology and Risk Factors

### 3.1. Definition

Patellar tendinopathy (PT), also known as jumper’s knee, is a persistent, load-related condition characterized by localized patellar tendon pain and impaired function during activities that load the knee extensor mechanism. Symptoms typically last for months to years. Histologically, the tendon is characterized by disorganized collagen fibers, altered extracellular matrix and proteoglycans, and neovascularization. Classic inflammatory cell infiltration, however, is not consistently present, and contemporary conceptualizations view tendon pathology as a continuum in which degenerative and inflammatory mechanisms may coexist and shift over time rather than as a simple degenerative-versus-inflammatory dichotomy [18]. Based on this pathological understanding, the term “patellar tendinopathy” has been widely adopted in clinical practice and reviews to distinguish the condition from the older term “patellar tendinitis,” which presupposes inflammation as the default mechanism. Repetitive loading of the patellar tendon, whether from jumping, cutting maneuvers, or other cyclic directional changes, is a well-recognized risk factor for patellar tendinopathy, although the condition is not sport-specific and develops in only a proportion of exposed athletes.

### 3.2. Epidemiological Surveys

A meta-analysis by Nutarelli et al., encompassing 28 studies and over 28,000 participants, reported an overall prevalence of approximately 18% (18.3%) among athletes, with rates heavily concentrated in jumping sports: 24.8% in volleyball, 20.8% in basketball, and 6.1% in soccer [1]. An earlier Norwegian survey of elite athletes by Lian et al. reported the same pattern [2].

#### 3.2.1. Epidemiology in Adults

In adults, the sport-specific prevalence of patellar tendinopathy follows a pronounced gradient: the higher the concentration of jumping load in a given sport, the higher the prevalence. The Norwegian elite athlete survey by Lian et al. reported a prevalence of 44.6% in volleyball, 31.9% in basketball, and less than 10% in endurance sports [2]. The meta-analysis by Nutarelli et al. extended this gradient from elite to non-elite populations, finding no significant difference between elite and non-elite basketball (18.9% versus 19.7%) [1], a finding that suggests competition level may not be a major driver of the observed gradient, although the absence of a statistically significant difference does not exclude a modest effect. At the population level, the observed gradient in adult risk appears to be primarily associated with the sport-specific loading profile rather than with competition level per se, although the available data cannot exclude a modest additional contribution of competition level.

Sex differences show a consistent trend, but the conclusions remain limited. Nutarelli et al. reported a higher prevalence in males (17% versus 11.2%), but the difference was not statistically significant [1]. In addition, as Mondini Trissino da Lodi et al. reported, most published treatment and prognostic data are drawn from male-dominated cohorts, and female athletes have been consistently underrepresented in this field [6]. To date, however, only one longitudinal study in a female gymnastics cohort has addressed this gap [19]. Thus, based on the current evidence, while prevalence is numerically higher in males, the true epidemiological profile of female athletes remains unclear.

#### 3.2.2. Epidemiology in Adolescents

The sport-specific prevalence gradient in adolescents mirrors that of adults, although prevalence varies by sport and is not uniformly lower than in adults. The prevalence is 26.6% in elite female gymnastics [19], 19.0% in competitive youth basketball [20], and 13.4% in elite youth soccer [21]. A three-year prospective observational study by Cassel et al. further showed that the incidence of patellar tendinopathy among adolescent elite athletes was 13%, compared with only 1% for Achilles tendinopathy over the same period [22], suggesting a stronger association of jumping-sport loading with patellar than with Achilles tendinopathy during growth.

The prevalence in adolescents is not evenly distributed and appears to be more common during the growth spurt. Longitudinal data from Harris et al. confirmed that proximal patellar tendon structural changes on ultrasound correlate linearly with maturity offset, a measure independent of chronological age [23]. Cencini et al. further localized the emergence of hypoechoic changes to the peak height velocity period and thereafter [24], suggesting that onset risk may vary according to developmental stage rather than chronological age. During this same developmental period, training volume among adolescent athletes may increase from approximately 5 to more than 15 h per week [22], potentially creating a mismatch between external loading and tendon adaptation, although this proposed mechanism has not been established directly. This population is epidemiologically unique in that the window of growth-related structural vulnerability coincides with a sharp increase in external load.

### 3.3. Risk Factors

Evidence on modifiable risk factors is generally weak, although a few findings have been replicated in independent cohorts. The systematic review by Sprague et al., covering 31 studies, constitutes the highest level of evidence available in this section and concluded that no single modifiable factor is strongly supported by evidence. Evidence for reduced ankle dorsiflexion range, diminished hamstring and quadriceps flexibility, and increased jump training volume was all rated as limited or conflicting [25]. Against this background, two factors have been replicated in independent cohorts. Backman et al. reported that restricted ankle dorsiflexion was significantly associated with increased risk of onset in elite adolescent basketball players [26]. However, this association has not been replicated consistently: the same systematic review rated the overall evidence for reduced ankle dorsiflexion as limited or conflicting [25], and, to our knowledge, no trial has yet demonstrated that modifying ankle range of motion reduces injury risk. In a separate prospective analysis, Mersmann et al. found that elevated patellar tendon strain during maximal voluntary contraction predicted subsequent symptoms in adolescent athletes, and suggested that this effect may reflect an imbalance between muscle strength gains and tendon stiffness adaptation, although the between-group difference in stiffness was not statistically significant [27]. A longitudinal observation by Visnes et al. showed that, among adolescent athletes who remained asymptomatic, quadriceps tendon thickness increased by 7% to 11% over the study period while patellar tendon thickness showed no corresponding change [28], suggesting that the adaptive capacity of the adolescent patellar tendon during development may lag behind the strength gains of the quadriceps and the structural adaptation of the quadriceps tendon. Mechanical risk factors show consistent trends but findings are reported inconsistently across studies. The systematic review by Harris et al. identified 37 jump-landing biomechanical variables but could not perform a quantitative synthesis [29]. Tayfur et al. subsequently included 16 studies and conducted a meta-analysis, finding that only reduced ankle dorsiflexion range of motion was consistently associated with PT (moderate-quality evidence), whereas most sagittal-plane kinematic and kinetic parameters, including vertical ground reaction force, peak knee and hip angles, and trunk kinematics, showed no significant relationship with PT. The PT group exhibited lower knee power and work during landing [30]. The same study noted that the absence of high-quality prospective studies precludes current evidence from supporting landing biomechanics as a causal predictor of PT. Overall, the evidence base for modifiable risk factors remains weak, limiting the development of individualized prevention strategies based on risk stratification. In addition, most individual risk-factor studies did not adjust for a common set of covariates, such as training exposure and prior injury, and cross-sectional associations may be confounded; the high prevalence of asymptomatic tendon abnormalities further complicates interpretation of screening studies.

## 4. Clinical Diagnosis and Imaging Assessment

### 4.1. Clinical Assessment

Clinical diagnosis remains the mainstay of patellar tendinopathy diagnosis. There is international consensus on terminology, defining patellar tendinopathy as persistent patellar tendon pain and loss of function related to mechanical loading [31]. Considerable consensus also exists on the defining clinical features of the condition [9]. However, no consensus has yet been established on standardized diagnostic criteria, particularly for imaging-based assessment, and no definitive clinical practice guideline currently exists [5]. In the absence of a diagnostic gold standard, diagnosis is based on history, physical examination, and functional assessment. The characteristic presentation consists of load-related pain and local tenderness at the patellar apex, with limitation in jumping, landing, and cutting. The first clinical challenge is to establish that the patellar tendon is the source of the symptoms [9]. Specific hallmark features are pain localized to the inferior pole of the patella and load-related pain that increases with the demand on the knee extensors, notably in activities that store and release energy in the patellar tendon; these features can be elicited and objectively assessed with clinical tests such as repeated single-leg squats, resisted knee extension, and repeated calf raises [9]. The Victorian Institute of Sport Assessment-Patella (VISA-P) is the most widely used quantitative outcome measure. Because it is primarily a patient-reported outcome measure rather than a diagnostic instrument, scores should be interpreted alongside, and compared with, the findings of a focused physical examination when establishing a diagnosis of patellar tendinopathy. A systematic review by Korakakis et al. reported that its reliability is supported by moderate-quality evidence and its construct validity by high-quality evidence, but content validity is supported only by very-low-quality evidence [7]. Morgan et al. proposed a preliminary screening framework integrating risk factors, VISA-P, and pain provocation tests, but the evidence base is limited to six studies [32]. Tendinopathy severity assessment-patellar (TENDINS-P), a new patellar tendinopathy-specific patient-reported outcome measure (PROM) introduced in 2026, has undergone content validation using the Consensus-based Standards for the selection of health Measurement Instruments methodology. It includes 14 items across three subdomains: pain (general), function, and pain (with loading) [8]. However, content validity represents only the first step of instrument validation; its construct validity, reliability, and responsiveness have yet to be established, and its clinical role therefore remains to be determined.

Because patellar tendinopathy is only one of many potential diagnoses producing anterior knee pain, the differential diagnosis should be considered explicitly [9]. Beyond the inferior pole of the patella, tendinopathy may also affect other parts of the knee extensor mechanism, such as the quadriceps tendon; the infrapatellar (Hoffa) fat pad can be a source of anterior knee pain; and patellofemoral-related pain, generally located diffusely around the patella compared with the typically localized inferior pole, may be the cause of anterior knee pain among jumping athletes [9]. In adolescents, Osgood–Schlatter disease and Sinding–Larsen–Johansson syndrome should be considered on the basis of age, activity, and tenderness at the tibial tuberosity or patellar pole.

### 4.2. Imaging Assessment

Imaging may supplement clinical assessment when diagnostic uncertainty remains or when alternative causes of anterior knee pain are suspected. Ultrasound is widely used because it is relatively accessible, inexpensive, portable, and capable of real-time and Doppler assessment of tendon thickness, echogenicity, and neovascularization. These characteristics make it practical for dynamic examination and, when clinically indicated, serial assessment. However, ultrasound is operator-dependent; acquisition and interpretation may vary, and structural abnormalities are also common in asymptomatic athletes. MRI provides higher soft-tissue contrast and a broader assessment of the extensor mechanism and adjacent intra-articular, osseous, and soft-tissue structures. It may therefore be useful when ultrasound findings are inconclusive or when an alternative or coexisting diagnosis is suspected. Its limitations include greater cost, lower accessibility, and the absence of real-time dynamic assessment. Importantly, the greater anatomical detail provided by MRI should not be interpreted as evidence of greater diagnostic or prognostic accuracy; in available prospective data, conventional MRI features did not predict clinical outcomes after exercise therapy [33].

Direct evidence regarding the diagnostic performance of history, physical examination, ultrasound, and MRI in patellar tendinopathy remains limited. Interpretation is constrained by the absence of a consistent independent reference standard, variation in imaging definitions and thresholds, and the frequent presence of structural abnormalities in asymptomatic tendons. Most studies have examined associations with subsequent symptoms or treatment outcomes rather than the sensitivity and specificity of these assessments for establishing a diagnosis. Consequently, no validated imaging-based diagnostic or prognostic algorithm is currently available, and the choice of modality should be guided by the specific clinical question rather than by an assumption that either ultrasound or MRI provides a definitive diagnosis or prognosis.

## 5. Treatment Approaches

The treatment literature is unevenly distributed across intervention categories and often draws on overlapping primary trials. The sections below therefore prioritize evidence syntheses and use individual trials to clarify direct comparisons, protocols, or newer findings rather than as independent confirmation.

Exercise therapy is the most extensively studied intervention and may be considered a reasonable first-line clinical approach, although the certainty of its comparative effectiveness remains low. Evidence for injections, physical modalities, and surgery is more limited or inconsistent. Key treatment studies and their designs are summarized in Table 1.

### 5.1. Exercise Therapy

Exercise therapy is widely used as a first-line intervention for patellar tendinopathy, but the evidence supporting its comparative effectiveness and any specific loading protocol remains limited in quantity and certainty. Mendonça et al. rated the evidence for conservative-treatment comparisons as very low to low certainty [34], while the Cochrane review by Lopes et al. found that none of the exercise comparisons across seven RCTs was supported by high-certainty evidence [35]. Network meta-analyses did not identify an adjunct that was clearly superior to exercise alone [5,15]. However, the absence of demonstrated superiority should not be interpreted as proof of exercise effectiveness or equivalence between interventions, particularly because these syntheses draw on a small and substantially overlapping group of primary trials. Exercise may nevertheless be considered a reasonable first-line option because it is non-invasive, can be progressively adapted to symptoms and functional capacity, and has shown improvement within several direct clinical studies, although the certainty of its comparative benefit remains low. The trials below clarify the available comparisons but do not establish the superiority of any single loading mode.

#### 5.1.1. Progressive Loading and Heavy Slow Resistance

Evidence syntheses comparing exercise modes have not established the superiority of progressive loading, HSR, or eccentric exercise with a high degree of certainty. The systematic review by Malliaras et al. found no clear difference between HSR and eccentric training in short-term VISA-P improvement, although greater patient satisfaction and some favorable structural changes were reported with HSR [10]. Subsequent network evidence on loading modes is limited to a mixed-comparison analysis by Li et al. based on seven small trials, in which moderate slow resistance exercise and concentric training had the highest probability of being the most effective interventions for VISA-P improvement (25% each) and eccentric exercise the lowest (1%) [16]. The authors noted that heavy slow resistance was more suited for long-term improvement in knee function, but also that the interventions could not be directly compared, which they identified as a significant limitation of the analysis. Because the network combined few, small trials, some of which were also included in the conventional systematic reviews discussed above, these patterns should not be interpreted as demonstrating that any loading mode is superior.

The primary trials underlying these syntheses provide further detail on the relevant head-to-head comparisons. In the JUMPER trial, Breda et al. randomized 76 patients with chronic symptoms to progressive tendon-loading exercise or a fixed eccentric exercise protocol. At 24 weeks, the progressive-loading group had higher VISA-P scores and lower pain during exercise [11]. A 13-point threshold has been used in some previous studies, but a universally validated Minimal Clinically Important Difference (MCID) for the VISA-P is not available. The reported 9-point between-group difference should therefore be interpreted cautiously, and its clinical importance remains uncertain. A post hoc analysis of the same trial found that reductions in tendon stiffness over 12 weeks were associated with improvements in VISA-P scores, whereas baseline stiffness did not predict the 24-week outcome [48]. As this was a secondary analysis of the same trial population, it provides exploratory mechanistic context rather than independent confirmation of the clinical effect. Moreover, associations between structural and clinical changes should not be interpreted as demonstrating that changes in tendon mechanical properties mediated the clinical response.

The three-arm RCT by Kongsgaard et al. included 39 participants and directly compared corticosteroid injection, eccentric training, and HSR [49]. All three groups improved at 12 weeks without a statistically significant between-group difference, but the corticosteroid group subsequently deteriorated, whereas improvements were maintained in both exercise groups at six months. HSR was also associated with greater patient satisfaction and changes in collagen turnover [49]. This trial contributed to subsequent evidence syntheses and should therefore not be regarded as evidence independent of those reviews.

Taken together, progressive loading and HSR have produced favorable findings in some direct comparisons, but the available evidence is insufficient to establish that either approach is consistently superior to other loading protocols. Structural measures provide information about tendon adaptation but should not be equated with clinical effectiveness because structural and symptomatic changes do not necessarily correspond. Selection of a loading approach may therefore also take account of symptom response, patient preference, equipment availability, and the feasibility of progressive load adjustment.

#### 5.1.2. Isometric Contractions

The available evidence suggests that isometric exercise may have a role in short-term symptom modulation, but it has not been shown to be superior to other contraction modes as a longer-term rehabilitation strategy. At the secondary-evidence level, the systematic review by Lim et al. assigned a Grade A recommendation to isometric exercise for pain reduction [36]. This rating was based largely on a small number of primary studies, including the trials described below, and should be interpreted within the review’s own grading framework and in light of the limited and inconsistent evidence base.

In an initial small study, Rio et al. reported that a single bout of isometric exercise produced immediate analgesia lasting for more than 45 min, accompanied by reduced cortical inhibition measured using transcranial magnetic stimulation [60]. A subsequent four-week in-season trial reported greater immediate analgesia with isometric than isotonic contractions and found that the early pain response was associated with functional improvement at four weeks [50]. However, a later study with a larger sample by Holden et al. found a smaller acute analgesic effect and no significant difference between isometric and isotonic exercise [61]. These studies form part of the evidence summarized in the corresponding reviews and should not be interpreted as an additional independent body of evidence.

Overall, isometric exercise may be considered as an optional short-term pain-management strategy, particularly when pain limits loading or sporting participation. The evidence does not establish that it reliably reduces pain in all patients or that it can replace a progressive loading programme, and variability in individual response should be acknowledged.

#### 5.1.3. Eccentric Training

Eccentric exercise has historically been a central component of patellar tendinopathy rehabilitation, but comparative evidence has not established its superiority over other loading modes. The network evidence is summarized in Section 5.1.1; the primary study below adds information on outcomes beyond VISA-P scores.

More recent primary evidence has examined outcomes beyond VISA-P scores. Fendri et al. reported improvements in static and dynamic postural control, quadriceps strength, and pain following targeted eccentric training in athletes with patellar tendinopathy [51]. However, this individual study did not directly evaluate subsequent injury incidence or sport-performance outcomes. Its findings therefore do not establish that eccentric training reduces future injury risk, improves athletic performance, or is superior to progressive loading.

Eccentric exercise remains a possible loading option, but the current evidence is insufficient to support its preferential use over progressive loading or HSR. Potential effects on injury prevention and sport performance require confirmation in prospective comparative studies specifically designed to assess these outcomes.

#### 5.1.4. Loading Parameters

Evidence concerning specific loading parameters is derived mainly from individual trials, with relatively limited secondary synthesis. The available studies have generally not detected statistically significant differences between the protocols compared, although their sample sizes, statistical power, and designs limit firm conclusions and do not establish equivalence.

Two trials by Agergaard et al. examined load magnitude and training frequency. No statistically significant differences in clinical outcomes or tendon structure were found between training at 90% and 55% of one-repetition maximum or between programmes performed once and three times per week at 12 weeks [12,52]. Hjortshoej et al. compared low-load blood-flow-restriction training with conventional HSR and found improvements in pain, function, and quadriceps morphology in both groups, without a statistically significant between-group difference; these improvements were maintained at 52 weeks [13]. Ruffino et al. likewise found no statistically significant between-group difference between inertial flywheel training and HSR [53]. Broader evidence has associated inertial flywheel training with improvements in strength, power, and tendon adaptation in physically active populations [64], but evidence from those populations should not be treated as direct evidence of clinical effectiveness in athletes with patellar tendinopathy. Moreover, because the trial by Ruffino et al. was not designed as an equivalence or non-inferiority trial, its null between-group finding does not establish that the two interventions are equally effective.

In a randomized crossover trial involving individuals with patellar tendinopathy, including non-athletic participants, Toustrup et al. detected no immediate difference in pain across loading intensities or exercise sequences [14]. This short-term finding does not rule out symptom aggravation with repeated high-intensity loading or establish longer-term safety and effectiveness in athletic populations.

Overall, the available trials do not identify a single optimal combination of loading intensity, frequency, or modality. Loading parameters may therefore need to be adapted to individual symptoms, capacity, treatment goals, and access to equipment, while recognizing that this approach is based partly on clinical practicality rather than evidence of equivalence among protocols.

#### 5.1.5. Exercise Interventions in Adolescents

The loading-mode comparisons discussed above were conducted predominantly in adults, and evidence concerning treatment of established patellar tendinopathy in adolescents remains limited. At the secondary-evidence level, the systematic review by Cairns et al. identified only three small RCTs in adolescents, all judged to be at high risk of bias, and found insufficient evidence to determine whether any particular exercise intervention was superior [65]. These trials represent the limited primary evidence underlying the review and should not be counted as a separate, additional body of evidence.

The 12-month cohort study of 207 adolescent female gymnasts by Steinberg et al. found that 9.7% of participants with patellar tendinopathy recovered, while 36.2% of the cohort developed patellar tendinopathy during follow-up [19]. These observational findings illustrate the persistence and incidence of the condition in this specific population but do not evaluate the effectiveness of an exercise intervention and may not be generalizable to adolescent athletes in other sports. Load management and symptom-guided return-to-sport guidance have shown promise in preliminary studies, but the evidence base for large-scale clinical application has yet to be established.

#### 5.1.6. Prevention and Tendon Adaptation in Adolescents

Several preliminary studies have examined prevention or tendon adaptation in adolescent athletes rather than treatment of established patellar tendinopathy. These studies address a distinct clinical question and should not be used as direct evidence for the effectiveness of treatment in symptomatic adolescents.

Mersmann et al. integrated functional high-load exercise into the regular training of adolescent handball players. By the end of the season, all participants in the intervention group were pain-free, whereas approximately 30% of the control group experienced clinically relevant worsening of pain [66]. Domroes et al. used an individualized loading protocol targeting approximately 6% patellar tendon strain and reported increased knee-extensor strength and patellar tendon stiffness, together with smaller fluctuations in peak tendon strain during the season, in the intervention group compared with controls [67]. Bittencourt et al. implemented an individualized prevention programme based on preseason assessment in elite adolescent volleyball and basketball players and reported an estimated risk in the intervention group of approximately half that observed in the control group [68].

These primary studies evaluated different populations, interventions, and outcomes and should not be interpreted as independent confirmation of a single preventive strategy. Their relatively small samples and other methodological limitations also restrict the strength and generalizability of the findings. Although individualized loading programmes show preliminary potential to influence symptoms, tendon adaptation, or injury risk, the current evidence is insufficient to identify a protocol that can be routinely recommended. Further controlled studies should distinguish prevention from treatment, use clinically relevant outcomes, and determine whether changes in tendon structure or mechanical properties translate into a lower incidence of symptomatic patellar tendinopathy.

### 5.2. Injection Therapies and Topical Agents

Injection therapies comprise clinically distinct interventions with different mechanisms and evidence bases. Most injection trials also incorporated structured exercise, making the injection-specific effect difficult to isolate. The findings are therefore presented separately for platelet-rich plasma (PRP), corticosteroid, hyaluronic acid, polidocanol, and cell-based interventions; topical glyceryl trinitrate (GTN) is included as a non-injection adjunct.

#### 5.2.1. Platelet-Rich Plasma

PRP is the most extensively studied injection intervention in patellar tendinopathy, but evidence syntheses have not established a consistent or sustained benefit. In the systematic review and meta-analysis by Barman et al., PRP was associated with more favorable outcomes than ESWT in head-to-head comparisons, but no significant difference was detected between PRP and other injection therapies [37]. The systematic review by López-Royo et al. suggested that outcomes may vary according to injection volume and the duration of accompanying exercise: PRP doses exceeding 4 mL combined with exercise lasting more than six weeks were associated with more favorable outcomes, whereas lower doses were not [38]. These subgroup findings should be interpreted cautiously and do not establish an optimal PRP protocol. In the network meta-analysis by Chen et al., leukocyte-rich PRP was associated with greater improvement than control in VISA-P scores (mean difference, 13.22 points; 95% CI, 2.37 to 24.07) and pain (mean difference, −1.87; 95% CI, −3.28 to −0.46) [39]. However, the limited network and uncertainty of the underlying evidence preclude conclusions regarding clinical superiority.

These syntheses incorporate much of the earlier primary evidence, including studies by Filardo et al., Dragoo et al., and Vetrano et al. [55,56,62]. Those studies are therefore not treated here as additional independent evidence. They are relevant because they illustrate why conclusions have varied: Filardo et al. reported an improvement in sports activity after PRP combined with physiotherapy [62]; Dragoo et al. found greater VISA-P improvement with leukocyte-rich PRP than with dry needling at 12 weeks, but not at 26 weeks, with both groups undertaking standardized eccentric exercise [55]; and Vetrano et al. reported a between-group difference favoring PRP over focused ESWT at 12 months [56]. These comparisons differed in their controls, rehabilitation programmes, PRP preparations, and follow-up periods.

A more recent multicenter RCT by Herrero et al., published after the earlier reviews, found no statistically significant additional benefit of leukocyte-poor PRP when added to dry needling and eccentric exercise at 12 or 26 weeks [57]. PRP preparations also vary in leukocyte content, platelet concentration, activation protocol, injection volume, and number of applications, further limiting comparisons across studies. Thus, PRP may be associated with earlier improvement in some settings, but its independent and sustained benefit beyond exercise or other concurrent treatment remains uncertain.

#### 5.2.2. Corticosteroid Injection

Corticosteroid injections are intended primarily to reduce pain and inflammatory activity rather than to promote tendon adaptation. In the network meta-analysis by Chen et al., corticosteroid injection was associated with less VISA-P improvement than control (mean difference, −23.00 points; 95% CI, −36.73 to −9.27), whereas the difference in pain was not statistically significant (mean difference, 0.80; 95% CI, −3.48 to 5.08) [39]. This estimate arose from a small network containing limited direct evidence and should not be interpreted as definitive proof of harm or inferiority.

The three-arm RCT by Kongsgaard et al., which contributed to the broader evidence base, found that improvement following corticosteroid injection was not maintained at six months, whereas improvement was maintained in the exercise groups [49]. This trial is discussed to clarify the time course of the direct comparison and should not be counted as evidence independent of the subsequent synthesis. Current findings raise concern about the durability of improvement after corticosteroid injection, but the small evidence base does not support a precise estimate of either benefit or harm.

#### 5.2.3. Hyaluronic Acid

Evidence for hyaluronic acid is sparse and of very low certainty. In the network meta-analysis by Challoumas et al., hyaluronic acid combined with eccentric exercise was associated with greater short-term VISA-P improvement than eccentric exercise alone (mean difference, −25 points; 95% CI, −49 to −2) [5]. The estimate was based on limited primary evidence and should be interpreted cautiously because of imprecision and reliance on the network assumptions. Since hyaluronic acid was administered alongside exercise, the available comparison does not determine its effectiveness as an independent treatment. Evidence concerning sustained outcomes is also insufficient.

#### 5.2.4. Polidocanol

Polidocanol sclerotherapy targets neovascularization and therefore differs mechanistically from PRP, corticosteroid, and hyaluronic acid. In the network meta-analysis by Wang et al., polidocanol was associated with greater VISA-P improvement than a no-injection control at six months [41]. However, the estimate was based on a limited number of contributing studies, and long-term outcomes were not available. The current evidence therefore suggests a possible medium-term effect in the comparisons studied but does not establish an independent or sustained clinical benefit.

#### 5.2.5. Cell-Based Interventions

Cell-based approaches comprise heterogeneous products rather than a single intervention. In the network meta-analysis by Wang et al., tenocyte-like cells were associated with greater VISA-P improvement than a no-injection control at six months, but the estimate was derived from limited evidence and longer-term outcomes were unavailable [41]. The systematic review by Kader et al. found no statistically significant between-group difference in imaging outcomes among studies classified as Level 1 or Level 2 evidence, whereas favorable functional outcomes were reported mainly in studies with greater methodological limitations [40]. Differences in cell source, preparation, dose, and delivery further restrict comparison across studies. These findings remain preliminary and do not establish the clinical effectiveness of cell-based interventions.

#### 5.2.6. Topical Glyceryl Trinitrate

GTN is a topical agent rather than an injection therapy, but it is considered here as another adjunct to exercise-based treatment. In the network meta-analysis by Challoumas et al., GTN combined with eccentric exercise was among the interventions receiving more favorable probability rankings for short-term pain, but the difference from moderate slow resistance exercise was not statistically significant (0.3 VAS points; 95% CI, −5.2 to 5.8), and the certainty of evidence was low or very low [5]. This ranking should not be interpreted as proof of clinical superiority.

The network estimate was based on limited primary evidence, including the placebo-controlled RCT by Steunebrink et al. [54]. In that trial, both the GTN-plus-eccentric-exercise and placebo-plus-eccentric-exercise groups improved, but no statistically significant between-group difference was detected in VISA-P scores or pain. A small number of participants receiving GTN reported mild skin rash [54]. Because this trial is part of the evidence summarized in the network meta-analysis, it does not constitute separate confirmatory evidence. The available evidence does not determine whether GTN provides a clinically meaningful benefit beyond exercise alone.

### 5.3. Physical Modalities and Adjunctive Measures

Physical modalities and adjunctive measures have been studied less extensively than exercise and injection therapies. The available evidence is summarized below for ESWT, taping, and straps.

#### 5.3.1. Extracorporeal Shockwave Therapy

ESWT is the most extensively studied physical modality for patellar tendinopathy. Earlier reviews suggested possible benefit, but more recent syntheses have generally found little or no additional benefit over sham treatment or exercise alone. Mani-Babu et al. reported favorable findings across several lower-limb tendinopathies, although patellar-tendinopathy-specific evidence was limited [42]. Korakakis et al. subsequently found, with moderate evidence, that focused ESWT was not superior to sham treatment at 3 to 6 months [43], while Charles et al. reported negligible effects on pain and function with low- to moderate-certainty evidence [44]. Network meta-analyses also found no clear additional benefit, including when ESWT was added to eccentric exercise [5,15]. A direct trial did not demonstrate clinical superiority of focused over radial ESWT, but its null result does not establish equivalence [58]. Protocol and comparator heterogeneity prevents a conclusion that ESWT is ineffective in every context; nevertheless, current evidence does not support its routine use as a first-line or stand-alone intervention.

#### 5.3.2. Taping and Straps

Patellar straps and athletic taping are widely used in clinical practice, but existing evidence supports only short-term symptom relief rather than modification of the underlying pathology. De Vries et al., in a randomized controlled trial, compared the effects of a patellar strap, athletic tape, and placebo tape on pain in patients with patellar tendinopathy. On the single-leg decline squat, patellar strap and sports tape significantly reduced pain versus control (14 mm, *p* = 0.04 and 13 mm, *p* = 0.04), but not versus placebo; during sports, strap, tape and placebo all reduced pain in the short term [63]. A randomized controlled trial by Ferreira et al. further demonstrated that adding dynamic taping to progressive tendon-loading exercise did not produce significant additional functional benefit [59]. Taping and straps can serve as adjunctive tools for short-term symptom management but should not replace other rehabilitation interventions.

### 5.4. Surgical Treatment

When conservative treatment has failed, surgery may be considered in selected patients with persistent symptoms after an adequate trial of conservative management. The Cochrane review by Dan et al. identified only two randomized controlled trials meeting inclusion criteria. The only trial directly comparing surgery with eccentric exercise found no statistically significant between-group difference in pain, function, or return-to-sport rate at 12 months, and one quarter of the exercise group crossed over to surgery due to unsatisfactory results [45]. Dan et al. concluded that surgery is chosen because conservative measures have been exhausted, not because evidence supports its superiority [45]. The systematic review by Mueller et al. included 15 studies, of which only two were randomized trials and the remainder were case series. Pooled results showed large improvements in postoperative VISA-P and pain scores, but between-study heterogeneity precluded meta-analysis [46]. No statistically significant difference in success rates has been found between open and arthroscopic surgery [47]. Overall, reported improvements after surgery are derived predominantly from observational studies and uncontrolled case series. In the absence of sham-controlled trials, the magnitude of the procedure-specific effect remains uncertain.

## 6. Long-Term Prognosis and Return to Sport

**Figure 1:**
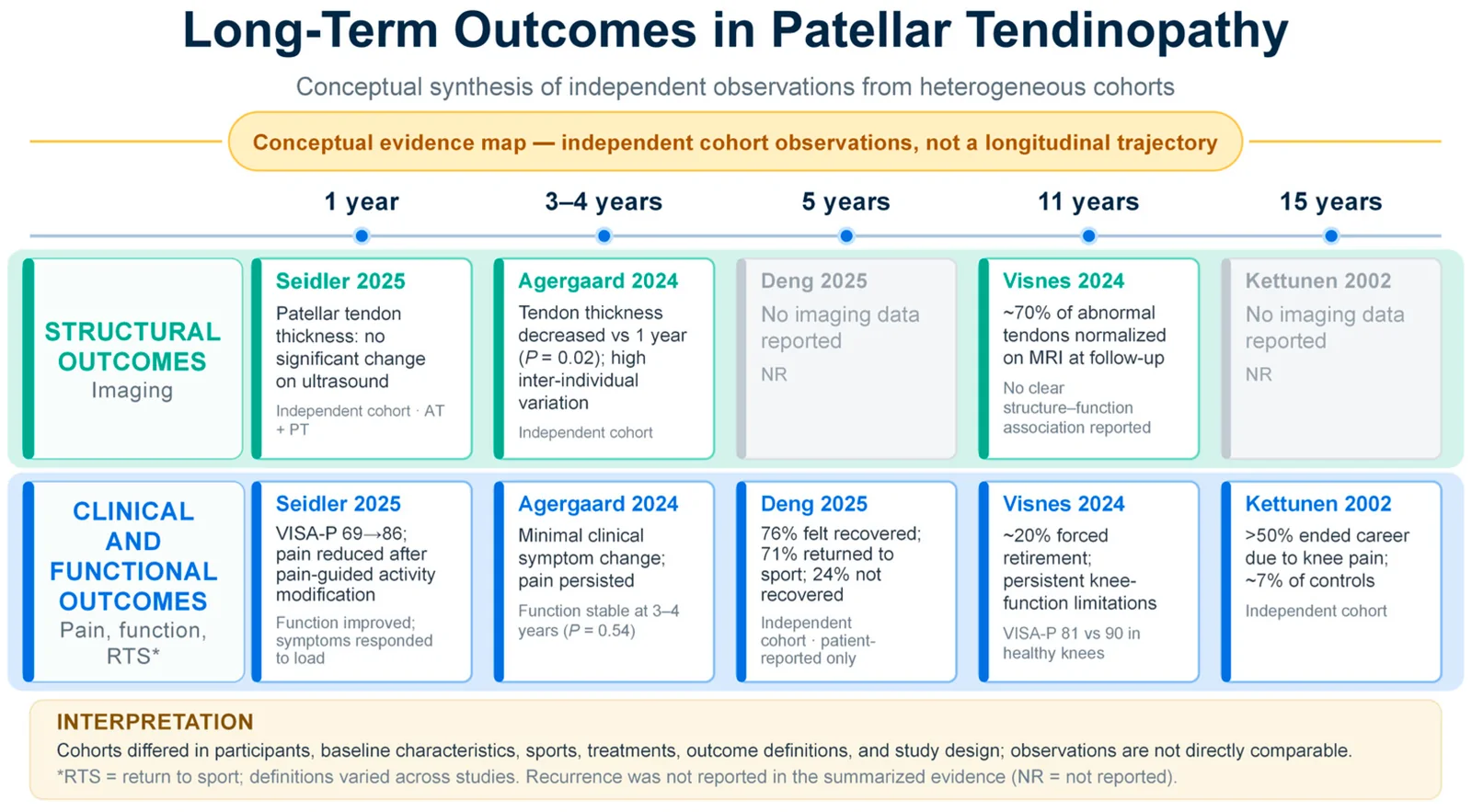
Long-term structural and functional observations in patellar tendinopathy (1–15 years), based on Seidler et al. [69], Agergaard et al. [70], Deng et al. [71], Visnes et al. [72], and Kettunen et al. [3]. This figure is a conceptual synthesis of discontinuous follow-up data from separate cohorts rather than an evidence-derived model or a longitudinal trajectory measured within a single sample; findings at each time point derive from different studies with differing populations, treatments, and outcome measures.

Return to sport is a major concern for athletes, but available studies examine separate cohorts at discontinuous follow-up points from 1 to 15 years. At one year, Seidler et al. reported improvement in pain and VISA-P scores after pain-guided activity modification, without significant structural change in the patellar tendon [69]. At 3 to 4 years, Agergaard et al. found persistent pain despite reduced tendon thickness, with substantial individual variation [70]. At five years, Deng et al. observed that most participants had returned to or exceeded their pre-injury ideal level of sport despite residual pain [71]. Over 11 years, Visnes et al. found persistently lower knee function and substantial career impact among affected volleyball players, even though many baseline structural abnormalities had normalized [72]. At 15 years, Kettunen et al. reported that more than half of athletes with jumper’s knee had ended their careers because of knee problems [3]. Across these cohorts, symptoms, function, participation, and tendon structure did not change in parallel. Figure 1 presents these observations as a conceptual evidence timeline.

Tayfur et al. developed the first exploratory recovery prediction model for patellar tendinopathy in 128 jumping athletes (C-statistic = 0.77) [17]. The model identified symptom response to exercise, training duration, subjective restfulness after sleep, baseline severity, previous time loss, and concurrent tendon problems as prognostic factors, with concurrent tendon problems showing the strongest adverse association. Sleep and training exposure may be modifiable, whereas baseline severity, previous time loss, and concurrent tendon problems are better viewed as prognostic characteristics. External validation is still required, and recurrence remains poorly documented because long-term studies have focused on persistent symptoms, functional limitation, and career termination rather than recurrence rates [3,72].

### Assessing Return-to-Sport Readiness

Validated criteria for return-to-sport readiness in patellar tendinopathy do not exist. Consistent with the 2016 international consensus framing RTS as a criterion-based continuum [4], decisions may consider four functional domains: (1) pain response during and after progressive loading [14,50,60]; (2) patient-reported function, commonly assessed with the VISA-P [7]; (3) strength and jump-landing capacity, including repeated single-leg squats and resisted knee extension [9]; and (4) sport-specific tolerance to the graded reintroduction of jumping, landing, and cutting tasks [4]. Psychological readiness may complement these assessments. In the absence of validated thresholds, progression should be individualized to symptoms, previous activity level, load tolerance, and psychological readiness rather than treated as a single pass-fail decision.

## 7. Imaging–Clinical Discordance

### 7.1. Imaging–Symptom Relationship in Adults

The discordance between imaging and symptoms is a recurring theme in patellar tendinopathy. In clinical practice, imaging is rarely used in isolation; the relevant question is therefore how much weight imaging findings should carry when they conflict with symptoms, rather than whether imaging has any clinical role. This discordance can be described across four dimensions: epidemiological, prognostic, mechanical, and pain-localization. At the epidemiological level, the meta-analysis by McAuliffe et al. showed that baseline ultrasound abnormalities increased symptom risk approximately fourfold, yet the absolute proportion of abnormal tendons that developed symptoms was low [73]. At the prognostic level, the prospective analysis by Deng et al. of 76 athletes with patellar tendinopathy found that neither individual baseline imaging parameters (on ultrasound or MRI) nor the total number of abnormalities predicted changes in pain or function scores at 24 weeks [33]. At the mechanical level, the meta-analysis by Obst et al. confirmed that patellar tendinopathy does not exhibit the consistent alterations in strain, stiffness, and modulus that have been widely reported in Achilles tendinopathy [74]. At the pain-localization level, the systematic review by Sahota et al. noted that the site of self-reported pain often does not align with the most prominent lesion on imaging [75]. These findings are not isolated anomalies within the field. They converge on a consistent misalignment between structure and symptoms.

Positive predictive associations are nonetheless reported in specific populations: in adolescent athletes, semi-annual ultrasound surveillance showed that baseline hypoechoic areas and neovascularization predicted subsequent development of patellar tendinopathy [28]; greater tendon disorganization on ultrasound tissue characterization was associated with higher patellar tendon risk [76]; and in a large collegiate cohort, ultrasound abnormalities at the patellar tendon were far more common than at other sites, although they carried the lowest risk ratio for time loss from sport [77]. Imaging abnormalities have shown predictive associations in some populations and for selected outcomes, but their incremental prognostic value beyond clinical assessment remains uncertain.

The tendon pathology continuum model offers one framework for interpreting imaging–symptom discordance, but it is not the only relevant perspective. Under this model, pain may occur during the reactive, disrepair, or degenerative stages rather than increasing in parallel with structural deterioration [78]. Alternative explanations include pain sensitization and neuropathic-like symptoms, load-dependent modulation of nociception, and regional mechanical compensation within the tendon. Neuropathic-like symptoms have been reported in patients with chronic tendinopathy, although the patellar tendinopathy subgroup was small [79]. The rapid reduction in pain reported after a single bout of isometric loading [60] also suggests that symptom intensity can change without corresponding short-term reversal of tendon matrix abnormalities. From a mechanical perspective, disorganized regions may contribute less to load transmission while structurally intact regions bear a greater proportion of the applied load [18]. In addition, some imaging abnormalities may reflect adaptation or previous pathology rather than the current source of symptoms, and structural improvement does not consistently accompany clinical improvement [80]. These explanations are complementary rather than mutually exclusive, and none currently provides a complete or prospectively validated account of imaging–symptom discordance in patellar tendinopathy. Imaging findings should therefore be interpreted in relation to the clinical presentation, population, imaging feature, and outcome of interest, rather than being assigned a uniform diagnostic or prognostic meaning.

### 7.2. Imaging–Symptom Relationship in Adolescents

The imaging–symptom discordance in adolescent patellar tendons adds a layer of developmental specificity to the adult framework described above. As in adults, imaging abnormalities are common in asymptomatic adolescents: only two-fifths of elite adolescent ballet dancers with sonographic pathology experienced pain, and pathology onset showed no clear association with the growth spurt [81]. Cencini et al. likewise reported no significant difference in pain scores between hypoechoic and normal groups [24]. However, the degree of discordance and predictive value are even more uncertain in adolescents than in adults. Semi-annual ultrasound follow-up by Visnes et al. (*n* = 141) found that baseline hypoechoic areas and neovascularization significantly predicted subsequent development of patellar tendinopathy, though the authors noted that this effect may be more pronounced in younger athletes than in adults [28]. Yet Cassel et al., in a composite cohort of adolescent elite athletes from 14 sports, reached a different conclusion: baseline ultrasound changes did not predict onset, and between-sport differences did not reach statistical significance (*p* > 0.05) [22]. Longitudinal data from Steinberg et al. supplemented the structure-symptom relationship from a dynamic perspective: over a 12-month follow-up of 207 adolescent female gymnasts, the proportion of athletes showing fiber disorganization decreased among those who remained healthy or recovered, whereas it increased among those with persistent or new-onset symptoms [19]. Regarding the drivers of structural change, longitudinal data from Harris et al. confirmed that proximal patellar tendon structural changes on ultrasound correlate linearly with maturity offset rather than chronological age, and that tendons with pre-existing hypoechoic areas did not deteriorate structurally under sustained high load [23]. Van Ark et al. likewise reported that 41 volleyball players aged 16 to 18 showed no significant change in tendon ultrasound structure after five days of high-intensity competition [82]. These findings suggest that indicators of maturation may be associated with the occurrence of structural tendon changes during adolescence. However, the observational evidence cannot determine whether these changes primarily reflect maturation, cumulative loading, short-term loading fluctuations, or interactions among these factors. At the mechanical level, Mersmann et al. found that symptomatic adolescent athletes exhibited significantly higher patellar tendon strain than asymptomatic counterparts [27]. Taken together, structural changes in adolescent patellar tendons have been associated with indicators of maturation but remain inconsistently related to pain and subsequent symptom development.

### 7.3. Practical Implications

Because imaging abnormalities are common in asymptomatic athletes [73] and imaging findings converge incompletely with symptoms at every assessment time point, two practical implications follow. First, current evidence is insufficient to support routine imaging-based screening of asymptomatic athletes. Although baseline tendon abnormalities have been associated with subsequent symptom development in some cohorts, their incremental predictive value beyond clinical and functional assessment remains uncertain. Screening may also identify incidental abnormalities whose clinical significance and appropriate management are unclear [73,77]. Second, in symptomatic athletes, improvement or persistence of imaging findings should not by itself trigger changes in treatment, and incidental findings should not be used to steer athletes away from loading-based rehabilitation when clinical assessment indicates that it is safe. Although the risk of overdiagnosis associated with incidental imaging findings has not been quantified in this population, a symptom-and-function-led approach reduces this risk and is consistent with the evidence summarized above.

## 8. Conclusions and Future Directions

Patellar tendinopathy should be managed primarily as a clinical, load-related condition rather than an imaging-defined disorder. History, physical examination, symptoms, and function should anchor diagnosis and follow-up, with imaging used selectively rather than as an isolated measure of severity, treatment response, or return-to-sport readiness. Progressive loading remains a reasonable first-line treatment, but the available low- to very-low-certainty evidence does not establish the superiority of any single loading mode. Evidence for injections, ESWT, and surgery is limited or inconsistent and is often difficult to separate from the effects of concurrent exercise; these interventions therefore cannot be considered routine substitutes for structured rehabilitation.

Prognosis remains insufficiently defined. Long-term findings from separate cohorts suggest that symptoms, function, sports participation, and tendon structure may change at different rates, but combining these observations reconstructs only a conceptual recovery trajectory, not a prospectively validated natural history or an individual prediction model. Evidence is particularly sparse for adolescents and female athletes, and recommendations for these groups still rely substantially on extrapolation from adult, predominantly male samples.

Future studies should prioritize adequately powered multicenter trials and prospective cohorts with standardized diagnostic definitions, validated patient-reported and return-to-sport outcomes, and sufficiently long follow-up to capture recurrence and persistent limitations. Research should also determine when imaging adds clinically useful information beyond examination and quantify the consequences of incidental findings. Until stronger evidence is available, the clearest clinical message is to individualize progressive loading and guide decisions by the athlete’s symptoms, function, and load tolerance rather than by structural imaging findings alone.

## Figures and Tables

**Table 1:** Key treatment studies for patellar tendinopathy.

Key Treatment	Study	Design	Population	Intervention Versus Control	Outcome	Follow-Up	Key Finding
**Evidence syntheses (systematic reviews, Cochrane reviews, network meta-analyses)**							
Strengthening (various)	Mendonça 2020 [34]	systematic review	9 RCTs; PT patients, any sport/level	Conservative treatment versus minimal/invasive intervention	pain, function (GRADE assessed)	Short ≤ 3 months, mid/long > 3 months	All conservative comparisons had very low to low GRADE certainty; insufficient evidence to support one treatment over another (risk of bias and imprecision as reported by the source review)
Strengthening (various)	Lopes 2025 [35]	Cochrane systematic review	7 RCTs, *n* = 211; chronic PT, athletes	Strengthening exercise versus no treatment/glucocorticoid injection/surgery/stretching	pain, function, return to sport, adverse events	end of treatment to 12 months	Very low to low quality evidence; no significant difference for pain, function, or return to sport at any timepoint
Eccentric/HSR/Isometric	Li 2024 [16]	Network meta-analysis	7 RCTs; PT patients, any level	Eccentric versus isometric versus moderate versus heavy slow resistance	VISA-P, pain, function	4 weeks–12 months	No loading intervention showed clear superiority; probability rankings favored moderate slow resistance and concentric exercise (25% each) over eccentric exercise (1%), but were based on only 7 RCTs and should be interpreted cautiously.
Eccentric	Malliaras 2013 [10]	systematic review	32 studies; athletes and active individuals with Achilles/patellar tendinopathy	Comparison of multiple loading programmes (eccentric, eccentric-concentric/HSR, etc.); not a single comparison of isometric vs. isotonic	VISA-P/VISA-A, pain, function	4 weeks–12 months	No single loading programme was clearly superior; where trials compared loading protocols, no statistically significant between-group difference was detected, and none of these trials was designed to test equivalence
Isometric	Lim 2018 [36]	systematic review	15 studies; PT patients, mostly athletes	High-load isometric versus dynamic resistance	pain, function, strength	variable	The source review assigned Grade A evidence to short-term isometric analgesia and Grade B evidence to eccentric exercise; these ratings were not independently reassessed in this review.
PRP (various)	Barman 2022 [37]	systematic review with meta-analysis	8 controlled studies; PT patients with chronic symptoms	PRP injection versus non-PRP injection/extracorporeal shockwave therapy/no-active treatment	pain, function	short/mid/long term	PRP showed more favorable medium- and long-term outcomes than ESWT in limited head-to-head comparisons; no clear advantage over other injection therapies was established.
PRP/Dry needling/Fibroblast	López-Royo 2020 [38]	systematic review with meta-analysis	10 RCTs, *n* = 326; PT patients, any level	Multiple minimally invasive techniques (PRP, dry needling, cell therapy) combined with exercise versus other treatments	VISA-P, pain (dose-stratified)	short/long term	Exploratory subgroup findings favored PRP volumes > 4 mL with concurrent exercise > 6 weeks; no superiority, optimal protocol, or dose–response relationship was established.
PRP/Cortisone/HA/Aprotinin	Chen 2019 [39]	Network meta-analysis	11 RCTs, 430 patellar tendons; PT patients	Multiple nonsurgical treatments versus control (LR-PRP, corticosteroid, ESWT, dry needling, GTN, etc.; network meta-analysis)	VISA-P, pain	variable	LR-PRP reduced pain versus control (−1.87; 95% CI, −3.28 to −0.46). Corticosteroid was associated with less VISA-P improvement than control in the network estimate (−23.0), based on limited direct evidence; this was not definitive evidence of inferiority or harm.
Mesenchymal stem cells/Bone marrow concentrate/Stromal vascular fraction/Tenocyte	Kader 2021 [40]	systematic review	14 studies; PT/knee injury patients, any level	Cell-based therapy (mesenchymal stem cells/bone marrow concentrate/stromal vascular fraction/tenocyte-like cells) versus control	imaging outcomes, functional outcomes	variable	Insufficient high-quality evidence for cell-based therapy; most studies were level 3 or lower
Polidocanol/PRP/corticosteroid/high-volume image-guided injection	Wang 2025 [41]	Network meta-analysis	19 RCTs; PT patients, any sport/level	Various injection strategies (polidocanol, tenocyte-like cells, LP-PRP, high-volume image-guided injection, dry needling, corticosteroid, HA, etc.) versus non-injection conservative treatment	VISA-P, VAS pain	1–6 months	Polidocanol, tenocyte-like cells, and LP-PRP + high-volume image-guided injection (HVIGI) showed medium-term (3–6 month) VISA-P benefit; no intervention showed long-term benefit
ESWT/GTN/corticosteroid/PRP + Exercise	Challoumas 2021 [5]	Network meta-analysis	37 RCTs; patients with patellar tendinopathy	Multiple treatment modalities (exercise, ESWT, injection, etc.); ESWT versus sham ESWT, both + eccentric	VISA-P/VISA-A, pain, function	short/mid/long term	ESWT + eccentric not superior to sham ESWT + eccentric; no adjunct clearly superior to exercise alone
Exercise ± multiple adjuncts	Challoumas 2023 [15]	Network meta-analysis	68 RCTs; patients with PT/Achilles tendinopathy/greater trochanteric pain syndrome, any level	Exercise with or without adjuncts (GTN, ESWT, injection, etc.) versus exercise alone	VISA-P/VISA-A, pain, function	short/mid/long term	No conclusive evidence that any adjunct is superior to exercise alone
ESWT	Mani-Babu 2015 [42]	systematic review with meta-analysis	20 studies; lower limb tendinopathy patients (greater trochanteric pain syndrome + patellar + Achilles tendinopathy)	Focused ESWT versus placebo/conservative/eccentric	pain, function	short < 12 months, long ≥ 12 months	Limited/moderate evidence: ESWT superior to other nonoperative treatments and as effective as surgery for patellar tendinopathy, as reported by the source review
ESWT	Korakakis 2018 [43]	systematic review	31 studies (12 RCTs); lower limb tendinopathy (Achilles, patellar, GTPS, MTSS, proximal hamstring tendinopathy)	ESWT/ESWT + eccentric versus placebo/eccentric/conservative	pain, function	short ≤ 3 months, mid 3–12 months, long ≥ 12 months	Moderate evidence: ESWT not superior to placebo shockwave for pain or function in PT
ESWT	Charles 2023 [44]	systematic review and meta-analysis	multiple systematic reviews/meta-analyses; PT/Achilles tendinopathy/plantar fasciitis patients	ESWT/ESWT + eccentric versus placebo/conservative treatment	pain, function	short/long term	Low-to-moderate evidence: ESWT has negligible effect on PT pain and function
Open tenotomy/Arthroscopy	Dan 2019 [45]	Cochrane systematic review	2 RCTs, *n* = 92; patients with PT refractory to conservative care	Open surgical excision versus eccentric exercises; arthroscopic surgery versus sclerosing injection	pain, function, return to sport, satisfaction	12 months	Surgery was not clearly superior to eccentric training at 12 months (VISA-P 72.9 versus 65.7; RR 0.71, 95% CI 0.27–1.88 for no pain at 12 months); evidence of low certainty; 5/20 participants in the eccentric group crossed over to surgery
Open/Arthroscopic surgery	Mueller 2025 [46]	systematic review	15 studies, *n* = 485; PT patients, mostly athletes	Summary of surgical outcomes (open and arthroscopic surgical techniques)	VISA-P, pain, return to sport rate	variable	Postoperative VISA-P improved by mean 39.6 points; heterogeneity in surgical techniques and outcomes
Arthroscopy/Open surgery	Marcheggiani Muccioli 2013 [47]	systematic review	21 studies (median *n* = 24/study); PT patients, athletes and non-athletes	Open versus arthroscopic surgery	return to sport rate, pain, function, satisfaction	mean 32.5 months	Overall return to sport rate 78.5%, success rate 84.6%; no statistical difference between arthroscopic and open approaches
**Randomized controlled trials**							
Progressive tendon-loading	Breda 2021 [11]	RCT	*n* = 76; PT athletes 18–35 years, tendon-loading sports ≥ 3sessions/week	Progressive tendon-loading exercise versus eccentric	VISA-P (primary), pain, return to sport	24 weeks	Progressive tendon-loading exercise within-group Δ ≈ 28 versus eccentric Δ ≈ 18; adjusted between-group difference 9 (95% CI 1–16); *p* = 0.023
Progressive tendon-loading	Breda 2022 [48]	RCT	*n* = 76; PT athletes 18–35 years, same cohort as Breda 2021	Progressive tendon-loading exercise versus eccentric	VISA-P, tendon stiffness (shear-wave elastography)	12, 24 weeks	Baseline patellar tendon stiffness was not associated with clinical outcome after 24 weeks (*p* = 0.52); decreasing stiffness during exercise therapy was associated with better outcome (adjusted mean difference 10 kPa, 95% CI 4–15; *p* = 0.02 at 12 weeks, *p* = 0.01 at 12 and 24 weeks in the progressive tendon-loading group)
Eccentric/HSR	Kongsgaard 2009 [49]	RCT	*n* = 39; male patients with chronic PT, recreational athletes	Corticosteroid injection versus eccentric versus HSR	VISA-P, VAS pain, tendon mechanical properties, satisfaction	12 weeks treatment, 6 months follow-up	All three groups improved significantly from 0 to 12 weeks (VISA-P: CORT 64 ± 14 to 82 ± 19; ECC 53 ± 13 to 75 ± 3; HSR 56 ± 13 to 78 ± 18; *p* < 0.01); from 12 weeks to follow-up, VISA-P deteriorated in the corticosteroid group (82 to 64) but was maintained in eccentric (75 to 76) and HSR (78 to 86); relative VISA-P improvement from baseline to follow-up was greater in eccentric (54%) and HSR (65%) than corticosteroid (13%) (*p* < 0.05); HSR showed the highest treatment satisfaction at follow-up (73%)
Isometric	Rio 2017 [50]	RCT	*n* = 20; in-season jumping athletes ≥ 16 years, sub-elite volleyball and basketball	Isometric (80% MVIC) versus isotonic	VISA-P, NRS pain (immediate + 4weeks)	4 weeks	Isometric contractions produced significantly greater immediate analgesia than isotonic (mean change 1.8 ± 0.39 versus 0.9 ± 0.25; effect size d = 2.75; *p* < 0.001; AUC 26.00 versus 14.23); VISA-P improved in both groups over 4 weeks with no between-group difference (change 10.5 versus 11.5 points; *p* = 0.99), and neither group median exceeded the 13-point MCID (80 versus 84/100); week-1 analgesic response correlated with 4-week VISA-P change (r^2^ = 0.64)
Eccentric	Fendri 2026 [51]	RCT	*n* = 40 (30 analyzed); athletes from local volleyball, basketball, and handball teams	Progressive loading eccentric decline squat versus control (continued regular sports)	VISA-P, VAS pain, postural control, quadriceps strength	12 weeks	Eccentric decline-squat training increased VISA-P by 20.24 points (+34.9%; g = 1.42; *p* < 0.01) after the intervention and by 18.5% more than in controls (mean difference 12.20; g = 0.74; *p* = 0.04); VISA-P scores did not differ at baseline (*p* = 0.30); VAS pain decreased by 68.7% in the eccentric group (mean difference 3.07; g = 1.19; *p* < 0.01) and was 67.7% lower than in controls (mean difference 2.93; g = 1.10; *p* < 0.01); baseline VAS did not differ (*p* = 0.85); quadriceps MVIC increased by ~17% (99 N) after the intervention (*p* < 0.01 vs. controls)
HSR	Agergaard 2021 [12]	RCT	*n* = 44; male adults with chronic PT, various sports	HSR (90% 1RM) versus MSR (55% 1RM)	VISA-P, NRS pain, tendon structure (MRI parameters, shear-wave elastography, cross-sectional area), strength	12 weeks, 52 weeks	Both HSR and MSR produced significant within-group improvements (VISA-P: HSR 58.8 ± 4.3 to 70.5 ± 4.4 at 12 weeks and 79.7 ± 4.6 at 52 weeks; MSR 59.9 ± 2.5 to 72.5 ± 2.9), with no statistically significant between-group difference (group effect *p* = 0.57); within-group improvements (+11.7 and +12.6 points) were approximately 12 points.
HSR (dosing)	Agergaard 2026 [52]	RCT	*n* = 52; patients with chronic PT, various sports	Short restitution (3 exercise days/week) versus extended restitution (1 exercise day/week)	NRS pain, VISA-P, tendon structure, strength	12 weeks (to 52 weeks)	No statistically significant group or group-by-time effect was detected between extended restitution (1 exercise day/week) and short restitution (3 days/week); VISA-P improved over 12 weeks by 12.0 (SR) and 14.4 (ER) points, both approaching the minimal clinically important difference of 13 points; no significant group (*p* = 0.16) or interaction (*p* = 0.54) effect, with a significant effect of time (*p* < 0.0001); pain during physical activity, daily activity, and rest decreased significantly in both groups (*p* < 0.0001) with no group differences; muscular strength increased by 21% (SR) and 20% (ER), with no significant group difference (*p* = 0.43)
HSR/BFR	Hjortshoej 2025 [13]	RCT	*n* = 36; male athletes with patellar tendinopathy	Low-load blood-flow restriction (30% 1RM) versus HSR	VISA-P, NRS pain, quadriceps morphology, strength	12 weeks, 52 weeks	Both groups showed clinically relevant improvements in pain during single-leg decline squat (LS mean: LL-BFRT 3.9 to 2.2 at 12 weeks, 1.8 at 52 weeks; HSRT 4.2 to 2.2 at 12 weeks, 1.1 at 52 weeks; *p* < 0.0001) and in VISA-P, with no statistically significant between-group difference; the trial was not designed to test equivalence
HSR/Inertial flywheel	Ruffino 2021 [53]	RCT	*n* = 42; athletes with PT, soccer/volleyball/running/basketball	Inertial flywheel versus HSR	VISA-P, pain, function, strength	12 weeks	Both groups improved significantly in VISA-P from 0 to 12 weeks, with no statistically significant between-group difference (*p* = 0.506); the absolute between-group difference in VISA-P was only 0.7 (6 weeks) to 3.2 points (12 weeks), much lower than the MCID of 13 points; the trial was not designed to test equivalence
GTN	Steunebrink 2013 [54]	RCT (double-blind)	*n* = 33; patients with chronic PT, recreational athletes	GTN patch versus placebo patch (both + eccentric decline squat)	VISA-P, pain, satisfaction	24 weeks	GTN showed no significant additional benefit (VISA-P 75.0 versus 80.7 at 24 weeks; treatment × time *p* = 0.80)
PRP/Dry needling	Dragoo 2014 [55]	RCT (double-blind)	*n* = 23; athletes with PT	LR-PRP versus dry needling (both + standardized eccentric exercises)	VISA-P (primary, 12weeks), VAS pain, return to sport (26 weeks)	26 weeks	PRP accelerated VISA-P improvement at 12weeks (+25.4 versus +5.2, *p* = 0.02); no significant difference at 26 weeks
PRP/ESWT	Vetrano 2013 [56]	RCT	*n* = 46; athletes with chronic PT, elite and nonelite	PRP versus focused ESWT	VISA-P, VAS pain, strength	6 months, 12 months	Satisfactory outcomes increased progressively after PRP (47.8%, 82.6%, and 91.3% at 2, 6, and 12 months) and after ESWT (43.4%, 65.2%, and 60.8% at the same time points), with a significant between-group difference in patient satisfaction only at 12 months (*p* = 0.035)
PRP/Dry needling	Herrero 2024 [57]	Multicenter RCT	*n* = 31; patients with PT, 2 centers	Dry needling alone versus dry needling + LP-PRP (both + eccentric exercise)	VISA-P, VAS, return to sport, pain, function	26 weeks	LP-PRP did not add any additional improvement over dry needling alone: at 12 weeks, VISA improved 19.2 ± 15.9 (LP-PRP) versus 28.4 ± 19.4 (DN), and VAS pain decreased −1.58 ± 2.1 versus −2.8 ± 1.9; no significant differences at 12 or 26 weeks
Focused versus Radial ESWT	Van Der Worp 2014 [58]	RCT	*n* = 43 (57 tendons); patients with PT, various sports	Focused ESWT versus radial ESWT (both + eccentric)	VISA-P, pain	14 weeks	Both groups improved significantly on the VISA-P, with no statistically significant between-group difference (focused ESWT +15 points versus radial ESWT +9.6 points)
Dynamic Tape	Ferreira 2025 [59]	RCT	*n* = 48; subjects with patellar tendinopathy	Dynamic Tape + progressive load exercise versus sham tape + exercise	VISA-P, VAS pain, return to sport	12 weeks	Adding Dynamic Tape to progressive-load exercise produced no additional benefit over sham tape (no significant group-by-time interaction for pain [*p* = 0.472] or severity [*p* = 0.286]); both groups improved over time (pain intensity: dynamic 6.04 ± 1.55 to 1.50 ± 1.99; sham 5.67 ± 1.78 to 1.57 ± 1.77; severity: 53.12 ± 9.94 to 83.65 ± 13.01 versus 51.21 ± 16.92 to 75.82 ± 17.12), with no statistically significant between-group difference at week 12 (pain −0.1 [−1.0–1.0]; severity 7.8 [−0.7–16.4])
**Other designs (randomized crossover and prospective controlled trials)**							
Isometric	Rio 2015 [60]	Randomized crossover	*n* = 6; male volleyball athletes with PT	Isometric versus isotonic (single bout; immediate + 45 min)	VAS pain (immediate, 45 min), cortical inhibition (TMS)	immediate + 45 min	Isometric exercise immediately reduced SLDS pain from 7.0 ± 2.04 to 0.17 ± 0.41 (isotonic: 6.33 ± 2.80 to 3.75 ± 3.28; *p* < 0.001), and reduced cortical inhibition; the analgesic effect was sustained for at least 45 min, whereas isotonic exercise did not produce sustained relief
Isometric	Holden 2020 [61]	Randomized crossover	*n* = 21; athletes with PT, mean age 27.9	Isometric versus dynamic (high-load) resistance exercise (single session)	NRS pain (immediate + 45 min)	immediate + 45 min	Both exercises reduced pain immediately after the bout (mean −0.9, *p* = 0.028) but no between-group difference; isometric not uniquely analgesic
Resistance load comparison	Toustrup 2025 [14]	Randomized crossover	*n* = 14; individuals with patellar tendinopathy	6RM versus 10RM versus 14RM leg press	NRS pain (immediate during exercise)	immediate	No statistically significant between-group difference in pain during exercise between relative loads (NRS 4.5 ± 1.7 at 6RM, 4.5 ± 1.7 at 10RM, 4.6 ± 2.0 at 14RM; F(2,26) = 0.06, *p* = 0.942)
PRP	Filardo 2010 [62]	Prospective controlled	*n* = 31; patients with chronic refractory PT, elite/recreational	PRP injections + physiotherapy versus physiotherapy alone	EuroQol visual analog scale, Tegner Activity Scale, pain, function	6 months	PRP plus physiotherapy improved significantly more than physiotherapy alone in sports activity (Tegner score percentage improvement 39 ± 22% versus 20 ± 27%; *p* = 0.048) at 6 months; in the PRP group, EQ VAS improved from 52.7 ± 22.0 to 78.3 ± 13.3 at 6 months (*p* = 0.007), pain decreased from 6.6 ± 1.4 to 3.1 ± 1.2 (*p* = 0.02), and Tegner score improved from 3.7 ± 1.8 to 6.6 ± 2.4 (*p* = 0.001); EQ VAS, pain, time to recover, and satisfaction did not differ between groups (control: EQ VAS 50.6 ± 22.3 to 73.5 ± 26.3; pain 6.7 ± 1.5 to 3.7 ± 2.8)
Patellar strap/Tape	De Vries 2016 [63]	RCT (multicenter crossover)	*n* = 97; athletes with PT, elite/recreational, 61% male	Patellar strap versus sports tape versus placebo versus control	VISA-P, VAS pain (immediate + 2 weeks)	immediate + 2 weeks	Strap and tape reduced single-leg decline squat pain (14 mm/13 mm, *p* = 0.04 versus control)

Note: The Population column lists participant characteristics extracted from source PDFs; the Outcome column lists primary and secondary outcome measures reported in each study. For systematic reviews, the n value indicates the number of included studies and/or total participant sample size. Table 1 groups studies by evidence layer (evidence syntheses; randomized controlled trials; other designs), as bold text marks the sub-title rows that separate the three layers, and the design of each study is stated in the Design column. Effect sizes, confidence intervals, and risk-of-bias information are reported where stated by the source studies; certainty assessments reported by the cited syntheses, such as GRADE, are noted in the corresponding rows. VAS, Visual Analogue Scale; TMS, Transcranial Magnetic Stimulation; MVIC, Maximal Voluntary Isometric Contraction; VISA-A, The Victorian Institute of Sport Assessment-Achilles; MSR, Moderate Slow Resistance; RM, Repetition Maximum; BFR, Blood Flow Restriction; PRP, platelet-rich plasma; LR-PRP, leukocyte-rich PRP. Where a 13-point VISA-P threshold is mentioned, it reflects the value used or cited by the source study and should not be interpreted as a universally validated MCID.

## Data Availability

No new data were created or analyzed in this study. Data sharing is not applicable to this article.

## Abbreviations

The following abbreviations are used in this manuscript:

MRI	Magnetic resonance imaging
HSR	Heavy slow resistance
PT	Patellar tendinopathy
VISA-P	Victorian Institute of Sport Assessment-Patella
TENDINS-P	Tendinopathy severity assessment-patellar
PROM	Patient-reported outcome measure
RR	Relative risk
RTS	Return to sport
GRADE	Grading of recommendations, assessment, development and evaluations
RCTs	Randomized controlled trials
PRP	Platelet-rich plasma
ESWT	Extracorporeal shockwave therapy
GTN	Glyceryl trinitrate
NRS	Numerical rating scale
S-LANSS	Self-report Leeds assessment of neuropathic symptoms and signs
